# Genome-Wide Characterization and Development of Simple Sequence Repeat Markers for Molecular Diversity Analyses in Yellowhorn (*Xanthoceras sorbifolium* Bunge)

**DOI:** 10.3390/plants13192794

**Published:** 2024-10-05

**Authors:** Xiaoming Yang, Yuan Wang, Yuewen Yang, Tuya Shareng, Yukun Xing, Gaowa Bai, Zhongyu Xing, Yuanyuan Ji, Liling Liu, Gongxiang Cao

**Affiliations:** 1 Co-Innovation Center for Sustainable Forestry in Southern China, Nanjing Forestry University, Nanjing 210037, China; 2 Inner Mongolia Academy of Forestry Science, Hohhot 010021, China

**Keywords:** yellowhorn (*Xanthoceras sorbifolium* Bunge), genetic diversity, simple sequence repeat (SSR) marker, core collection

## Abstract

Yellowhorn (*Xanthoceras sorbifolium* Bunge) is a valuable ornamental, medicinal, and woody oilseed species that is indigenous to China. The breeding improvement of yellowhorn has been hindered by a lack of suitable markers and sufficient information regarding the molecular diversity of this species. In this study, we conducted a comprehensive analysis of the yellowhorn genome to characterize the simple sequence repeat (SSR) loci. A total of 4,007,201 SSRs were successfully identified. Among these markers, mono-nucleotide SSRs were most abundant in the genome, while the tri-nucleotide SSRs accounted for the highest proportion in coding sequences. The GO and KEGG function enrichment analysis revealed that most SSR loci in coding sequences were associated with potential biological functions. Additionally, we used 30 pairs of primers to amplify SSR markers to gain a better understanding of the genetic variation in yellowhorn germplasms. The average values of observed heterozygosity and polymorphism information content were 0.625 and 0.517, respectively. Population structure, phylogeny and principal component analyses identified two distinct subclusters. Furthermore, yellowhorn germplasms with the same geographical distribution tended to group together. Moreover, a total of 26 yellowhorn core collections, which accounted for approximately 14.94% of the total yellowhorn germplasms, effectively represented the genetic diversity of all original germplasms. Our findings not only unveiled the genetic diversity and population structure of yellowhorn germplasms but also investigated the yellowhorn core collection, which will serve as a strong basis for yellowhorn management and genetic improvement.

## 1. Introduction

Yellowhorn (*Xanthoceras sorbifolium*), a member of the Sapindaceae family, is an indigenous ornamental, medicinal, and oil tree species in China [1]. The seed kernel may contain up to 67% oil, notably rich in essential unsaturated fatty acids such as oleic acid and linoleic acid, which are crucial for dietary requirements [2]. Notably, the concentration of nervonic acid, vital for brain health and development, is approximately 3.04% in yellowhorn seed oil [3]. Xanthoceraside, a novel triterpenoid saponin isolated from yellowhorn husks, exhibits anti-cancer properties and holds potential for Alzheimer’s disease treatment [4]. Its therapeutic efficacy has contributed to its recent adoption as a medicinal ingredient. Additionally, it also holds high ornamental value and is extensively utilized in urban landscape construction due to its vibrant and colourful flowers during various developmental stages [5].

To effectively explore and utilize germplasms, assessing the genetic diversity of species is essential [6]. In general, elevated genetic diversity within species correlates with enhanced biological survivability, environmental adaptation, and ultimately facilitates genetic improvement, allowing for the selection of desirable agronomic characteristics. Conversely, low genetic diversity can lead to the eventual decline of a species, as it may lack necessary adaptation and viability [7]. Owing to overexploitation and environmental degradation, yellowhorn germplasms have significantly diminished in recent years, exacerbated by the expansion of intensive farming practices and the influence of climate change [8]. Recent research on yellowhorn primarily concentrates on its pharmacological activity, economic significance, and ornamental values [3]. Various molecular markers, such as random amplified polymorphic DNA (RAPD), amplified fragment length polymorphism (AFLP), simple sequence repeat (SSR), inter-simple sequence repeat (ISSR), and single nucleotide polymorphism (SNP), have been effectively utilized to investigate the genetic diversity of yellowhorn and promote the progression of molecular breeding in this species [1,9]. However, molecular assessment of yellowhorn germplasms has lagged behind, possibly due to the restricted accessibility of genetic or genomic resources for yellowhorn. Hence, a deep understanding of the genetic diversity of yellowhorn is instrumental in facilitating the exploitation of germplasms and contributing to ecological conservation.

SSRs, also known as microsatellites, are short repeat sequences that are widely distributed in genomes. As one of the most robust molecular markers, owing to their codominance and polymorphism characteristics, they have found extensive applications in investigating genetic diversity, facilitating marker-assisted breeding, elucidating phylogenetic relationships, and exploring molecular ecology in plants [10,11]. With rapid advancements in next-generation sequencing, thousands of SSRs in various species have been successfully developed based on whole genome data [12]. Several SSR markers were developed to facilitate the estimation of the genetic diversity and population structure of yellowhorn [13]. Based on the pairwise kinship coefficient derived from SSR markers among yellowhorn germplasms from different populations, a molecular marker-based conservation strategy framework for yellowhorn has been developed [14]. Combining SSR data with phenotypic information revealed a relatively low level of diversity and genetic variation among distinct yellowhorn populations. These results indicated that SSR markers were well-suited for exploring the genetic diversity of yellowhorn.

Although the release of high-quality yellowhorn genome has greatly facilitated the identification of molecular markers in genetic research [15], it has been infrequently employed in mining SSR markers to develop appropriate breeding programs for yellowhorn. Our research aimed to: (1) characterize SSR variation in the yellowhorn genome and develop polymorphic SSR markers; (2) unveil the genetic diversity and population structure of yellowhorn germplasms; and (3) identify core yellowhorn germplasms to enhance the yellowhorn molecular breeding process. The study will contribute to enhancing our understanding of the molecular diversity of yellowhorn and prove valuable for the effective utilization and conservation of yellowhorn germplasms.

## 2. Results

### 2.1. Characterization of SSRs in the Yellowhorn Genome

**Figure 1:**
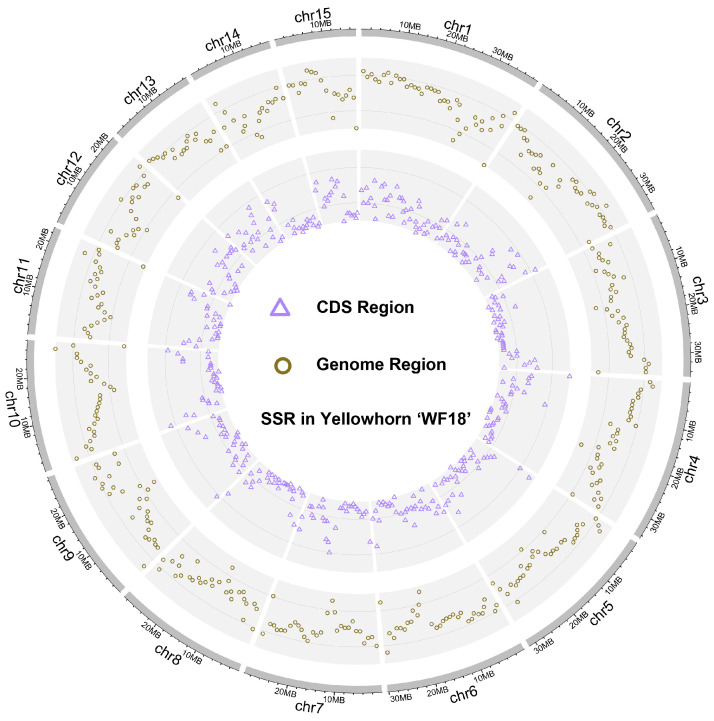
Genome-wide distribution of SSR markers on the yellowhorn ‘WF18’ chromosomes. The rings from the inner circle to the outer circle show SSRs distributed on the CDS and genome regions, respectively.

**Figure 2:**
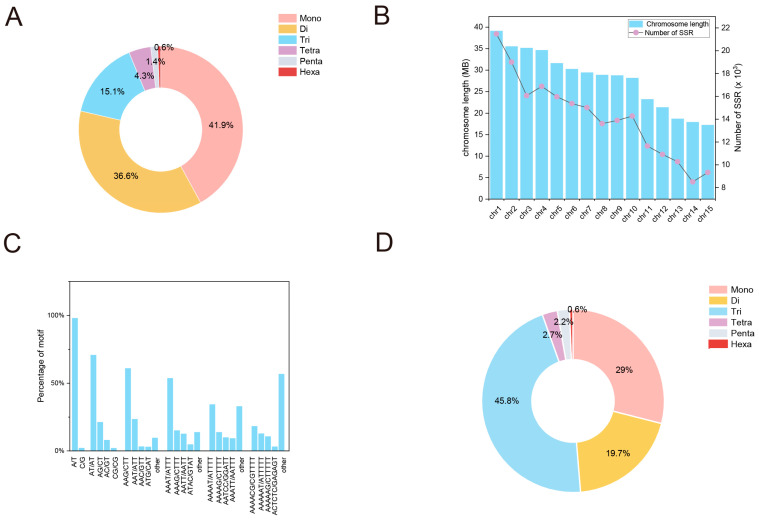
Characteristics of SSR loci in the yellowhorn genome. (**A**) Proportion of SSRs with different motif types in the yellowhorn genome regions. Different colours represented mono-, di-, tetra-, hexa-, penta-, and hexa-nucleotide repeats. (**B**) Correlation analysis between chromosome length and the number of SSRs in the yellowhorn genome. (**C**) Frequency of different motifs within the same types in the yellowhorn genome. (**D**) Proportion of SSRs with different motif types in the yellowhorn genome CDS regions.

In the yellowhorn (2n = 30) genome, a total of 212,270 SSR loci were identified, averaging 9781.28 SSRs per Mb. Each of the SSR loci was physically mapped onto 15 distinct chromosomes, as shown in Figure 1 and Supplementary Table S1. As the length and repeat times of the motif increased, there was a dramatic decrease in the number of SSR loci. Specifically, mono-nucleotide SSRs were the most numerous (89,032), followed by di-nucleotide (77,726), tri-nucleotide (32,138), tetra-nucleotide (9194), penta-nucleotide (2894), and hexa-nucleotide (1286) SSRs. The proportion of SSR loci for each type diminished with an increase in SSR length, with mono-nucleotide, di-nucleotide and tri-nucleotide SSRs having proportions of 41.9%, 36.6%, and 15.1%, respectively (Figure 2A). The distribution analysis revealed that chromosomes 1 and 15 had the highest and lowest numbers of SSRs, respectively (Figure 2B). Moreover, the quantity of SSRs on chromosomes exhibited a significantly positive correlation with the length of each chromosome (r = 0.988, *p* < 0.001). The frequency distribution of various SSR motifs in the yellowhorn genome was also analyzed (Figure 2C). Among these, the A/T motif was most prevalent (40.06%), followed by AT/TA for di-nucleotide (25.91%), AAG/CTT for tri-nucleotide (9.22%), AAAT/ATTT for tetra-nucleotide (2.32%), AAAAT/ATTTT for penta-nucleotide (11.71%), and AAAACG/CGTTTT for hexa-nucleotide (0.11%).

Among the identified SSR loci, a total of 4368 SSR loci were successfully minded from 22,406 coding sequences (CDSs) with a density of 134.34 SSRs/Mb (Figure 1). A total of 3293 CDSs, accounting for 14.70% of all CDSs, contained SSR loci. Among these CDSs, 746 CDSs had more than one SSR locus, accounting for 17.08%. Tri-nucleotide repeat motifs were the most abundant with a representation of 45.8%, followed by mono-, di-, tetra-, hexa-, and penta-nucleotide repeats at 28.9%, 19.7%, 2.7%, 2.2%, 0.6%, respectively (Figure 2D). The frequency distribution analysis of different SSR motifs in CDSs indicated the highest occurrence of A/T (28.57%) for mono-nucleotide, followed by AAG/CTT (11.17%) for tri-nucleotide, AG/CT (9.68%) for di-nucleotide, AAAT/ATTT (0.98%) for tetra-nucleotide, AAAAT/ATTTT (0.21%) for penta-nucleotide, and ACCTCC/GGAGGA (0.16%) for hexa-nucleotide SSRs. The major mono- to hexa-nucleotide repeats were A, AG, AAG, AAAT, AAAAT, and ACCTCC, with the A motif being the most abundant with a relative abundance of 579.82 bp/Mb, followed by AAG (267.86 bp/Mb) and AG (223.04 bp/Mb) motifs. However, there were fifty-six unique penta-nucleotide types, and each contained only one member. Our results indicated that there was no inverse relationship between SSR abundance and the number of motif repeats, and this trend was more conspicuous, especially for tri- and hexa-nucleotide SSRs.

### 2.2. Functional Annotation for the SSR-Associated Genes

**Figure 3:**
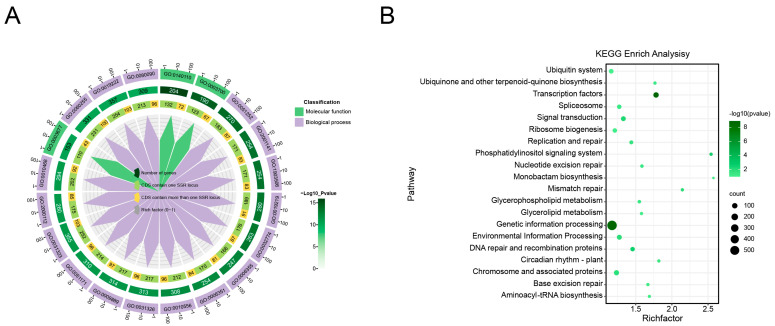
Functional enrichment analysis of SSR-containing genes in the yellowhorn genome. (**A**). GO annotation of the SSR-containing genes. Dark green, light green, and yellow colours represented the number of genes, CDS contained only one SSR locus, and CDS contained more than one SRR locus. (**B**). Enriched KEGG pathway among the SSR-containing genes. The x-axis and y-axis present the rich factors and KEGG pathways, respectively. Dot size signifies the number of unique genes, while dot colour denotes the q-value.

The CDSs containing SSR loci were annotated with the eggNOG database, encompassing GO and KEGG categories. The GO database provided annotations for 1244 genes, and the KEGG database annotated 1105 genes, respectively (Supplementary Tables S2 and S3). A total of 731 genes exhibited simultaneous matches in both databases. GO annotation revealed that CDSs containing SSR loci belonged to two categories, namely molecular function and biological process (Figure 3A). Based on the findings, CDSs containing SSR loci were primarily associated with the regulation of metabolic processes, with a focus on the metabolic process (357 genes), followed by the cellular metabolic process (355 genes), and the macromolecule metabolic process (331 genes). Interestingly, CDSs belonging to these three metabolic processes tended to contain more than two SSR loci. According to the KEGG database annotation, CDSs containing SSR loci were predominantly associated with genetic processing information (563 genes), followed by transcription factors (74 genes), and signal transduction (60 genes). Additionally, it was observed that genes in significant metabolic pathways, including glycerophospholipid metabolism (16 genes) and glycerolipid metabolism (16 genes), which were closely related to lipids, exhibited a preference for harbouring SSR loci (Figure 3B). Furthermore, CDSs within the transcription regulator activity category tended to possess more than one locus (54.55%), whereas those falling under the DNA binding domain were inclined to harbour only a single SSR locus (39.09%).

### 2.3. Development, Validation and Physical Mapping of Polymorphism SSR Markers

**Figure 4:**
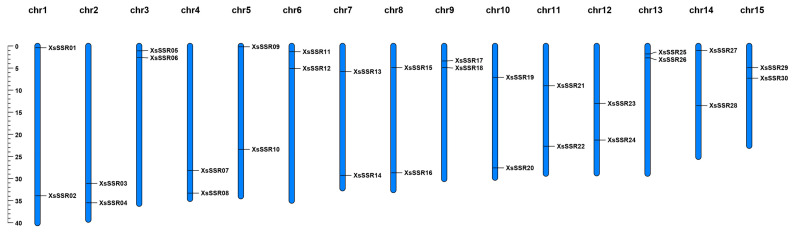
Chromosomal distribution of SSR markers utilized in this study.

A total of 160,571 primer pairs were successfully designed from the flanking regions of SSR loci on different chromosomes in the yellowhorn genome (Supplementary Table S4). Subsequently, one hundred primer pairs were randomly selected and synthesized for verification using six yellowhorn germplasms with distinct geographic origins. Consequently, 30 pairs of primers were identified to produce clear and polymorphic bands in different germplasms, establishing them as candidate polymorphic primers. These were then employed to evaluate the genetic diversity and population structure of all germplasms. Other primers were excluded due to nonspecific amplification and unexpected amplicon lengths. All identified candidate polymorphic SSRs exhibited uneven distribution across the 15 different chromosomes of the yellowhorn genome (Figure 4 and Supplementary Table S5).

### 2.4. Analysis of Genetic Diversity and Population Structure

Utilizing 30 pairs of polymorphic primers, we assessed the genetic diversity of yellowhorn germplasms (Table 1). In total, 234 alleles were amplified across all yellowhorn germplasms using 30 pairs of primers, resulting in a mean of 7.80 alleles per locus. Values of Na ranged from 5 to 11, while those of Ne varied from 2.092 to 6.712. For these loci, values of Ho varied from 0.319 to 0.776, while those of He ranged from 0.067 to 0.859. The averages for Ho and He were 0.563 and 0.689, respectively. Regarding the PIC values, out of the loci examined, 27 had values larger than 0.5, while the remaining 3 had values less than 0.5 but larger than 0.25. The average PIC value for the 30 pairs of polymorphic SSR markers was 0.634.

**Figure 5:**
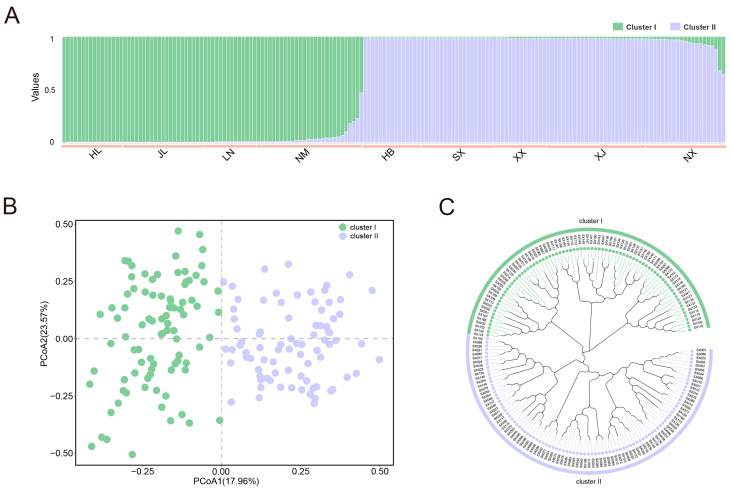
Genetic clustering of 174 yellowhorn germplasms with 30 pairs of SSR primers. (**A**) Population structure analysis of all yellowhorn germplasms with K = 2; (**B**) Principal component analysis (PCoA); (**C**) Phylogenetic tree analysis based on the genetic distances.

To gain a deep understanding of the genetic variation and population structure of yellowhorn germplasms, we further performed population structure, PCoA, and phylogenetic analyses. Based on the Bayesian clustering approach, we successfully inferred the population structure of all yellowhorn germplasms (Figure 5A). The results showed that the optimal number of clusters was identified using the more value of ΔK (K = 2) and all yellowhorn germplasms were divided into two obvious clusters (cluster I and cluster II) (Figure S1). There were 80 and 94 samples in clusters I and II, respectively. Furthermore, cluster I contained yellowhorn germplasms that mainly originated from northeast and northern China, while germplasms grouped together in cluster II were collected from northwestern China. Nearly all samples could be assigned into these two clusters, except for a few individuals that showed admixture characteristics. According to the correlation genetic similarity matrix, the derived scatter plots generated from the PCoA analysis clustered all yellowhorn germplasms into two obvious clusters (Figure 5B). The first and second coordinates accounted for 17.94% and 23.57% of the total variation, respectively. Similar to the results obtained from both population structure and PCoA analyses, the NJ tree constructed based on the Ne’s genetic distance revealed that all yellowhorn germplasms could also be divided into two major clusters (Figure 5C). All findings indicated the clustering of populations tended to be consistent with the geographical origin of each germplasm.

### 2.5. Establishment of Yellowhorn Germplasm Core Collection

**Figure 6:**
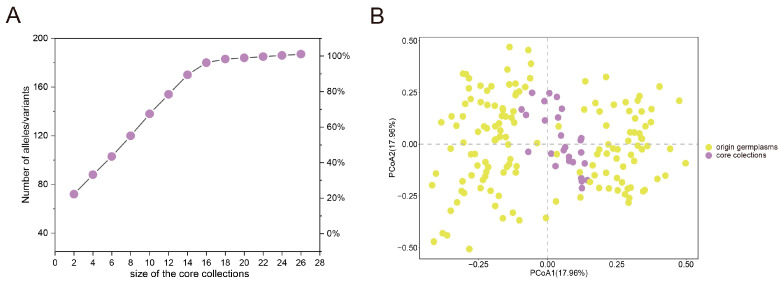
The genetic diversity of the yellowhorn core collections. (**A**) Identification of yellowhorn core collections based on the strategy of maximizing allelic diversity; (**B**) Principal coordinates analysis (PCoA) was performed on the core collections and compared with the original germplasms.

To investigate the core collection of yellowhorn germplasms, we delineated the minimum subset of core collections encapsulating the full spectrum of genetic variance within all germplasms detected with 30 SSR markers. The number of sampled alleles increased proportionally with the sample size according to the maximizing strategy until reaching a plateau with no substantial change when the sample size was 26 (Figure 6A). Consequently, we defined a set of 26 yellowhorn germplasms (14.94% of the total germplasms) as a core collection encapsulating the genetic diversity inherent to all germplasms. Comparative analyses of all germplasms against core collections using the Mann–Whitney U test revealed no significant genetic differences across various parameters, including Na, Ne, I, Ho, He, and PIC (Supplementary Table S6). Furthermore, the PCoA analysis was conducted to ascertain the representation accuracy of the genetic diversity of all germplasms as reflected by the core collections (26 yellowhorn germplasms), based on SSR marker data. The results indicated that nearly all core collections were grouped together in the middle part of the scatter plot, demonstrating an accurate representation of all germplasms (Figure 6B).

## 3. Discussion

Compared to crops, the breeding of perennial plants, particularly trees, is encumbered by long breeding cycles, a high level of heterozygosity, complex genome structure, and limited functional genomic information [12,16]. Molecular marker-assisted selections are considered an effective strategy to enhance breeding precision and abbreviate breeding cycles. With advancements in biotechnology, a growing number of high-quality tree genomes have been sequenced and made available, facilitating the identification of SSR markers across all genes in several tree species [16]. Although the high-quality yellowhorn genome is released [15], the lack of genome-wide identification of polymorphic SSR makers has significantly impeded the progress of molecular breeding and the cloning of genes associated with important agronomic traits. SSR markers closely linked to functional genes could aid in selecting individuals with favourable agronomic traits at an early growth stage, thereby expediting tree breeding efficiency [17]. Previous studies on yellowhorn genetic diversity relied on SSR markers derived from incomplete genome or expressed sequence tags [13,18]. In contrast, this study identified numerous SSR loci using the complete yellowhorn genome, which will advance both our understanding of genome structure evolution and support future molecular breeding efforts for yellowhorn.

The varied frequency and density of SSRs in the genome and CDS regions are attributed to various factors, including genome size, gene dimensions, structures, amounts, and the criteria employed for SSR mining [19]. The proportion of SSR loci within the CDS was 14.70%, similar to *Carya illinoinensis* [20], but less than in *Juglans regia* (22.3%) [21]. Apart from mono-nucleotide repeats, di-nucleotide repeats are often the most prevalent in the genomes of species. Coincidentally, we also observed that di-nucleotide repeats were most prevalent among all six motif types assessed in the yellowhorn genome. Moreover, the AT/AG motif category was the most frequent in the yellowhorn genome, aligning with prior research on *Punica granatum* [22]. However, tri-nucleotides were the predominant motif type in CDS regions, consistent with the report on *Carya illinoinensis* [20]. Since coding regions must maintain the reading frame, tri-nucleotide repeats are known to minimize the risk of frameshift mutations in coding regions and thus have minimal adverse effects on the integrity of the coding framework. Therefore, tri-nucleotide repeats are anticipated to be more frequent in CDS areas compared to other repeat types [23]. Among tri-nucleotides, AAG/CTT (11.17%) was found to be the most frequently occurring motif, aligning with findings in *Salix* and *Populus* [24]. Our results further confirmed that di-nucleotide repeats were the major repeat type in plant genomes, while tri-nucleotide repeats dominated in the CDS regions.

SSRs in CDSs have been extensively studied concerning human health, yielding a wealth of information about cancers and neurological disorders [25]. Likewise, comprehensive analyses of SSRs across a range of plants have uncovered the potential functions of numerous genes linked to key agronomic traits, including fruit type, skin and flesh colour, flowering time, shell hardness, and salt tolerance [12,16,26,27]. Based on the GO and KEGG function annotations, it emerged that certain CDSs containing SSR loci were integral to glycerophospholipid and glycerolipid metabolism, which are crucial to lipid metabolism. Yellowhorn, an indigenous oil-bearing woody tree, is extensively cultivated in northern China for its bioactive oil production. Diverse yellowhorn germplasms exhibited significant variation in oil content, with seed kernels possessing up to 67% oil, including nervonic acid, noted for its health benefits [28,29]. Presently, the breeding of yellowhorn prioritizes enhancing oil yield, particularly the nervonic acid concentration. A focus on the polymorphic CDSs containing SSR loci might shed light on the mechanisms of oil metabolism. Consequently, these SSR markers in CDS regions may serve as potent and cost-effective tools for genotypic fingerprinting and marker-assisted selection, facilitating a marked increase in selection efficacy for perennial woody species.

To advance yellowhorn breeding and fully exploit yellowhorn germplasms, a comprehensive survey of molecular diversity within yellowhorn germplasms was essential. SSR markers are widely used to reveal the genetic diversity of numerous species due to advantages such as cost-efficiency in development, widespread genomic distribution, and significant transferability and polymorphism [17]. Utilizing 30 primer pairs a total of 234 alleles and the average He value of yellowhorn were identified, which is consistent with prior outcomes from SSR marker-based genetic diversity assessments in yellowhorn [13,18,30]. In most cases, He was higher than Ho in yellowhorn, indicating a degree of heterozygote deficiency. Heterozygote deficiency could result from factors such as self-pollination, inbreeding depression, genetic drift, and limited gene flow [31,32]. Previous research has demonstrated high divergence and relatively low gene flow in 38 natural populations based on chloroplast SSR markers. Population isolation reflects the species’ habitat fragmentation and inbreeding depression [8]. Our results indicated heterozygote deficiency in yellowhorn germplasms covering the species’ natural range based on nuclear SSR markers. We inferred that factors such as habitat fragmentation and inbreeding depression were possible causes of the lower observed heterozygosity compared to the expected heterozygosity in yellowhorn. Additionally, heterozygote deficiency in yellowhorn would be attributed to silent alleles that fail to amplify via PCR, which could lead to erroneous assessments of parentage [33]. The PIC value is commonly utilized to assess a marker’s informativeness, with a threshold of 0.5 or higher signifying its robust capacity to discern polymorphisms at a distinct locus. In our study, the mean PIC value was 0.634, with a maximum value reaching 0.761, demonstrating that these SSR markers possessed substantial polymorphism, underscoring their significant potential in evaluating genetic variations across yellowhorn germplasms. The mean PIC value in our investigation was comparatively higher than that for previous results about yellowhorn germplasms [30,34]. Consequently, our findings, corroborated by previous studies, suggested that yellowhorn exhibited a considerable degree of genetic variation.

Combining the results from population structure, PCoA, and NJ analyses, all yellowhorn germplasms were separated into two distinct clusters, correlating with their geographic origins. As we know, geographic isolation resulting from various mountains, rivers, and plateaus can restrict pollen and seed dispersal among populations, leading to a scattered distribution across the species’ habitats [35]. Our findings revealed significant genetic differentiation between clusters I and II. Considering the distribution of yellowhorn germplasms examined in this study, several mountains and valleys constituted geographic barriers among these distributions, likely leading to significant genetic differentiation. Similarly, geographic isolation has also significantly influenced the distribution and genetic diversity of many species, including *Sophora alopecuroides* [36] and *Populus tomentosa* [37] in the northern part of China.

Core collections were strategically composed of a minimal set of samples designed to capture the broadest genetic diversity, which has attracted greater attention in molecular breeding programs [38]. Previous research has demonstrated that 5–20% of the same sample size could represent the genetic diversity of original collections [38]. A similar investigation on *Corylus avellana* [39] and *Ginkgo biloba* [40] with sampling ratios of 16.6% and 12.64%, respectively, showed allelic richness with origin collections. Twenty-six germplasms, comprising 14.94% of the original germplasm, were identified, yielding the highest level of allelic retention in yellowhorn. The established yellowhorn core collection will be utilized in future genome association studies and breeding programs. Further, we should use different data types including phenotypic data and molecular data to construct yellowhorn collections to improve the quality of core germplasm sets.

## 4. Materials and Methods

### 4.1. Plant Materials

A total of 174 yellowhorn germplasms were collected from the nine widely spread natural populations covering the majority of the geographical distribution of the species in China (Supplementary Table S7). Healthy and fresh leaves from yellowhorn germplasms were randomly selected, and different germplasms were separated by at least 1000 m to avoid similar genotypes.

### 4.2. Characterization of SSR Loci in Yellowhorn Genome

The genome of yellowhorn ‘WF18’ was downloaded from the genome database [15]. Coding sequences exhibiting alternative splicing were omitted according to the GFF annotation file criteria, ensuring that unique and non-duplicative sequences were deployed for analysis. Krait software (https://krait.biosv.com/en/latest/, accessed on 1 April 2023) [41] was utilized to scrutinize the occurrence of precise SSR repeats. Analysis consideration entailed mono- to hexa-nucleotide motifs, with prescribed minimum repeat units established at 12 for mono-nucleotides, and 6, 4, 4, 4, and 4 for di-, tri-, tetra-, penta-, and hexa-nucleotides, respectively. The physical distance between adjacent SSRs was set to more than 100 bp. The primer for each SSR locus was designed using Krait software. Primers were designed with the following parameters: a final product length of 110–230 bp, primer sizes ranging from 18 to 22 bp, with an optimal size of 20 bp, and an annealing temperature of 60 °C. The distribution of SSR density in the yellowhorn genome was shown using shinyCircos v 2.0 [42].

### 4.3. Functional Annotation of SSR-Containing Genes

To assess the potential function of CDSs containing SSRs, both CDSs and CDSs containing SSRs were subjected to BLAST analysis (E-value < 1 × 10^−5^) against a localized InterProScan database [43]. Functional enrichment analysis was then performed with the clusterProfiler package [44] in R software (v 4.3.0), identifying significant enrichment terms based on the criteria (*p*-value < 0.05, *q*-value < 0.05).

### 4.4. DNA Extraction and PCR Amplification

DNA from each germplasm was isolated from the leaves using the plant genomic DNA isolation kit (Magen, Guangzhou, China). For the identification of polymorphic markers, six germplasms from different geographical origins were selected for polymorphism screening. Potential polymorphic molecular markers with an even distribution in the yellowhorn genome were chosen as candidate test markers. PCR reactions were conducted, and PCR products were confirmed based on our previous research [36,40]. Polymorphic SSR markers were mapped to each chromosome based on their physical position using MapChart v2.2 software [45].

### 4.5. Genetic Diversity and Population Structure Analysis

Codominant genotypic data were presented as two columns per locus and alleles were coded numerically based on the required format in GenAlEx 6.5 [46] for further analysis. Genetic diversity parameters, such as effective allele number (Ne), allele number (Na), polymorphic information content (PIC), observed heterozygosity (Ho), expected heterozygosity (He), and Shannon’s information index (I), were computed employing GenAlEx v 6.5 and Arlequin v 3.5 [47].

Using a Bayesian model-based clustering algorithm, the population structure of yellowhorn germplasms was examined through STRUCTURE v 2.3 [48]. This assessment encompassed ten independent runs for each presumed number of clusters (K = 1 to 10), entailing an initial burn-in period of 10,000, followed by 100,000 Markov Chain Monte Carlo (MCMC) iterations, assuming an admixture model with independent allele frequencies. Structure Harvester software (http://taylor0.biology.ucla.edu/structureHarvester, accessed on 1 April 2023) guided the selection of optimal K values, indicating ΔK. The configuration of clusters for each K was harmonized using CLUMPP v 1.1.2 [49] and DISTRUCT v 1.1 [50] illustrated the aggregated output as bar charts for the most probable K. Complementary to STRUCTURE analysis, Principal Component Analysis (PCoA) and a Neighbor-Joining (NJ) phylogenetic tree, constructed on genetic distances, were both methodically applied, using GenAlEx v 6.5 and MEGA v 11 [51], respectively.

### 4.6. Identification of Yellowhorn Core Germplasms

Yellowhorn core germplasms were established based on diversity analyses conducted with CoreFinder v 1.1 (http://services.appliedgenomics.org/software/corecollections, accessed on 1 April 2023). Utilizing the M strategy coupled with a Las Vegas algorithm, an optimal sample ratio was automatically designated for the assembly of these core collections. We then evaluated the disparities between the established core collections and the complete germplasms pool using PCoA conducted via GenAlEx v 6.5.

## 5. Conclusions

In this study, we thoroughly characterized SSR loci in the yellowhorn genomes and identified a series of valuable polymorphic SSR markers. Some CDSs containing SSR loci had putative metabolic and biological functions, as indicated by functional annotation. A set of 30 SSR markers revealed a relatively high genetic diversity in 174 yellowhorn germplasms, which were further divided into two distinct clusters based on population structure, PCoA, and NJ analyses. A total of 26 core yellowhorn germplasms, accounting for 14.94% of the initial germplasms, were successfully identified, demonstrating their potential use in future breeding programs. Our results will not only lay the foundation for molecular evaluation but also facilitate the genetic improvement of yellowhorn germplasms via marker-assisted breeding.

## Figures and Tables

**Table 1:** Genetic information of yellowhorn.

Locus	Na	Ne	Ho	He	I	PIC
XsSSR01	9	3.039	0.516	0.651	0.623	0.552
XsSSR02	8	5.124	0.469	0.528	0.421	0.506
XsSSR03	10	6.712	0.594	0.721	1.201	0.734
XsSSR04	10	5.212	0.392	0.586	0.854	0.512
XsSSR05	9	5.562	0.452	0.523	0.875	0.525
XsSSR06	10	5.254	0.698	0.754	1.035	0.584
XsSSR07	9	4.589	0.582	0.685	0.784	0.574
XsSSR08	11	6.575	0.587	0.758	0.895	0.852
XsSSR09	7	4.258	0.457	0.762	1.324	0.721
XsSSR10	8	4.521	0.458	0.757	1.356	0.712
XsSSR11	8	5.311	0.412	0.623	1.023	0.582
XsSSR12	9	6.563	0.667	0.854	1.896	0.756
XsSSR13	7	4.268	0.428	0.665	1.101	0.683
XsSSR14	6	3.254	0.319	0.621	0.825	0.488
XsSSR15	7	4.332	0.682	0.768	1.524	0.635
XsSSR16	7	3.085	0.601	0.607	1.425	0.587
XsSSR17	6	2.092	0.582	0.520	1.058	0.487
XsSSR18	7	4.756	0.452	0.789	1.526	0.758
XsSSR19	5	2.636	0.467	0.622	1.223	0.478
XsSSR20	5	3.345	0.501	0.782	1.354	0.654
XsSSR21	9	5.369	0.745	0.827	1.582	0.752
XsSSR22	10	5.458	0.704	0.812	1.586	0.761
XsSSR23	7	4.528	0.751	0.778	1.526	0.723
XsSSR24	6	3.485	0.712	0.859	1.452	0.631
XsSSR25	8	6.012	0.776	0.842	1.821	0.758
XsSSR26	8	5.125	0.648	0.768	1.752	0.658
XsSSR27	9	4.231	0.425	0.605	1.205	0.561
XsSSR28	7	4.526	0.623	0.785	1.523	0.598
XsSSR29	7	5.127	0.705	0.759	1.652	0.658
XsSSR30	5	2.852	0.498	0.587	1.058	0.532
Mean	7.8	4.573	0.563	0.689	1.249	0.634

Note: Na: Number of alleles; Ne: effective number of alleles; I: Shannon’s information index; Ho: Observed heterozygosity; He: Expected heterozygosity; PIC: Polymorphism information content.

## Data Availability

All data are contained within the article and its Supplementary Files.

## Supplementary Materials

The following supporting information can be downloaded at: https://www.mdpi.com/article/10.3390/plants13192794/s1, Figure S1. Graph of estimated ΔK value for 174 yellowhorn germplasms with ΔK = 2; Table S1. Characteristics of SSR loci in the yellowhorn genome; Table S2. Characteristics of SSR loci in CDS regions; Table S3. CDSs containing SSR loci in the yellowhorn genome are successfully annotated with GO and KEGG databases; Table S4. Primer sequences for all SSR loci have been successfully designed; Table S5. Polymorphic primers used in genetic diversity analysis; Table S6. Mann–Whitney U Test (two-tailed) for the genetic diversity parameters of yellowhorn core collections and all yellowhorn germplasms; Table S7. Detailed information for 174 yellowhorn germplasms in this study.
