# Supplementary figures and images for: Genome-Wide Characterization and Development of Simple Sequence Repeat Markers for Molecular Diversity Analyses in Yellowhorn (Xanthoceras sorbifolium Bunge)
Source: Plants (Basel). 2024 Oct 5;13(19):2794. doi: 10.3390/plants13192794 (PMC11479088; doi:10.3390/plants13192794)

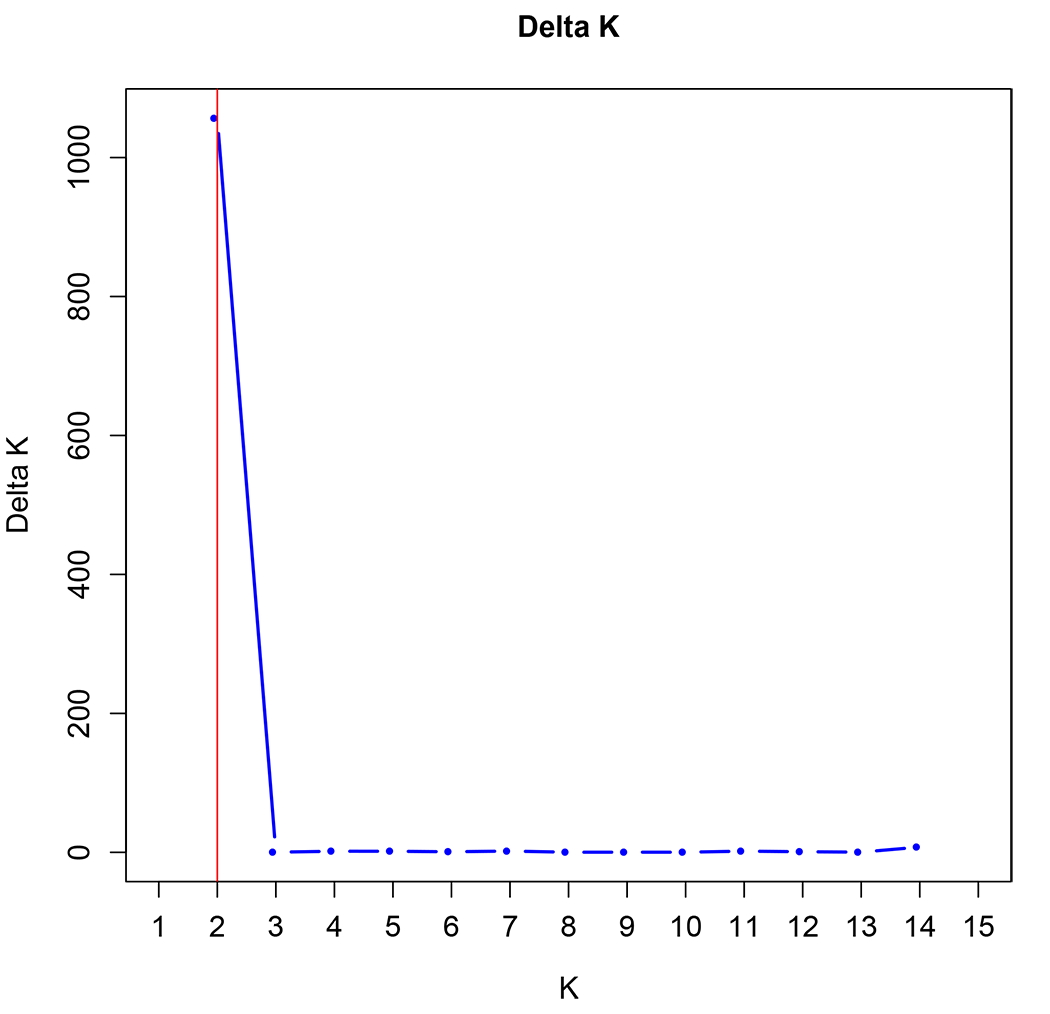

Supplement: Supplementary file 1 [file plants-13-02794-s001.zip › Figure S1.tif]
